# MARCKS contributes to stromal cancer-associated fibroblast activation and facilitates ovarian cancer metastasis

**DOI:** 10.18632/oncotarget.8726

**Published:** 2016-04-13

**Authors:** Zongyuan Yang, Sen Xu, Ping Jin, Xin Yang, Xiaoting Li, Dongyi Wan, Taoran Zhang, Sixiang Long, Xiao Wei, Gang Chen, Li Meng, Dan Liu, Yong Fang, Pingbo Chen, Ding Ma, Qinglei Gao

**Affiliations:** ^1^ Cancer Biology Research Center, Tongji Hospital, Tongji Medical College, Huazhong University of Science and Technology, Wuhan, Hubei 430030, China

**Keywords:** ovarian cancer, CAFs, MARCKS, senescence, Twist1

## Abstract

The Cancer Genome Atlas network has revealed that the ‘mesenchymal’ epithelial ovarian cancer (EOC) subtype represents the poorest outcome, indicating a crucial role of stromal cancer-associated fibroblasts (CAFs) in disease progression. The cooperative role of CAFs in EOC metastasis has long been recognized, but the mechanisms of stromal CAFs activation are still obscure. Therefore, we carried out an integrative analysis to identify the regulator genes that are responsible for CAFs activation in microdissected tumor stroma profiles. Here, we determined that myristoylated alanine-rich C-kinase substrate (MARCKS) was highly expressed in ovarian stroma, and was required for the differentiation and tumor promoting function of CAFs. Suppression of MARCKS resulted in the loss of CAF features, and diminished role of CAFs in supporting tumor cell growth in 3D organotypic cultures and in murine xenograft model. Mechanistically, we found that MARCKS maintained CAF activation through suppression of cellular senescence and activation of the AKT/Twist1 signaling. Moreover, high MARCKS expression was associated with poor patient survival in EOC. Collectively, our findings identify the potential of MARCKS inhibition as a novel stroma-oriented therapy in EOC.

## INTRODUCTION

High-grade epithelial ovarian cancer (EOC) remains the most lethal gynecological cancer and exhibits considerable heterogeneity [1]. Traditional histopathological classification of EOC into “serous,” “mucinous,” “clear cell,” and “endometrioid” subtypes was limited in guiding therapy decisions [2, 3]. To complement conventional histopathology, molecular classification based on large-scale gene profiling was carried out and enabled the discovery of several EOC subtypes [4–6]. Tothill et al. first reported six molecular categories in 285 EOC patients and found that tumors expressing a reactive stromal gene signature were associated with a poor prognosis [4]. More recently, The Cancer Genome Atlas (TCGA) project described four subtypes, namely, “immunoreactive,” “differentiated,” “proliferative,” and “mesenchymal,” in a cohort of 557 serous EOC patients [5]. A subsequent follow-up study determined that patients with the “mesenchymal” subtype presented the worst prognosis [7]. These newly emerged classification schemes based on molecular profiling facilitate our understanding of EOC heterogeneity and the development of personalized treatment strategies [8–10]. Moreover, this advantageous stratification emphasized the importance of tumor microenvironment, especially in terms of the stromal infiltrating components in EOC patients.

The most prominent cell types in the tumor microenvironment are the cancer-associated fibroblasts (CAFs), which primarily contributed to the assignment of the “mesenchymal” cluster [11, 12]. CAFs are heterogeneous populations that include myofibroblasts and reprogrammed variant normal tissue derived cells such as fibroblasts and endothelial and mesothelial cells in EOC [13–15]. Generally, CAFs support cancer cells through both cell-to-cell contact interactions and soluble factors via secretion of cytokines, chemokines and ECM (Extra-cellular matrix) components [16–18]. The cooperative role of CAFs in EOC cell proliferation, adhesion and metastasis has long been recognized, but the mechanisms involved in stromal CAF activation are still largely unknown [18–20]. Previous studies have emphasized that perpetual activation of stromal CAFs is indispensable for tumor expansion [14–19]. Therefore, understanding the molecular profile of activated CAFs could help in targeting this major accessory of tumor microenvironment. Recent molecular investigation has identified a “stromal-response” signature that predicts poor prognosis [21] and defined a “reactive stroma signature” characterizing primary chemoresistance in EOC [22]. Although previous studies sought to characterize specific ovarian tumor stromal genes in a compartmentalized fashion, the samples used were whole tumor specimens, which cannot exclude the interference of the epithelial compartment. Meanwhile, the recently discovered stromal signature genes are more likely to be downstream functional genes than the upstream regulator genes. These factors prompted us to explore the underlying regulators that control the active CAF signature in pure tumor stromal tissues.

The emergence of specialized microdissected stroma profiling data allows the identification of tumor stroma gene signatures, as well as the potent regulator genes controlling stroma activation [18, 23–25]. To identify the regulators of stromal CAF activation, we employed the most widely accepted marker of the CAF phenotype—αSMA, which constitutes a stress fiber system bridging communication between CAFs and the ECM [26]. Thus, we carried out an integrative analysis of microdissected stromal gene profiles of EOC and invasive breast tumors [18, 25]. Among the genes identified, myristoylated alanine-rich C-kinase substrate (MARCKS) was found to be highly associated with αSMA expression in EOC tumor stroma, and was notably overexpressed in tumor stroma of both ovarian and invasive breast cancer. MARCKS, originally identified as a major target of protein kinase C (PKC), is a key regulatory molecule regulating actin dynamics [27]. Recently, it has been shown to play a fundamental role in mediating chemoresistance of breast and lung cancer [28, 29]. There are limited studies examining MARCKS in EOC metastasis. Although MARCKS has been reported to promote fibroblast migration [30], the role of MARCKS on CAF traits and the underlying mechanism involved is not well understood.

This study demonstrates elevated stromal expression of MARCKS along with OC advancement, and shows that MARCKS sustains the CAF features through suppression of cellular senescence and maintenance of AKT signaling. Suppression of MARCKS attenuates CAF activity and their tumor-supporting role in 3D organotypic culture and an OC murine xenograft model. A meta-analysis of a total of 2970 serous OC expression profiles confirmed MARCKS as a prognostic factor of poor patient outcome. Our results address the role of MARCKS in the tumor stroma as a pivotal regulator of CAF activation. Thus, MARCKS could be an attractive target for stroma-oriented therapy in EOC patients.

## RESULTS

### Overexpression and significance of MARCKS in tumor stromal fibroblasts

**Figure 1:**
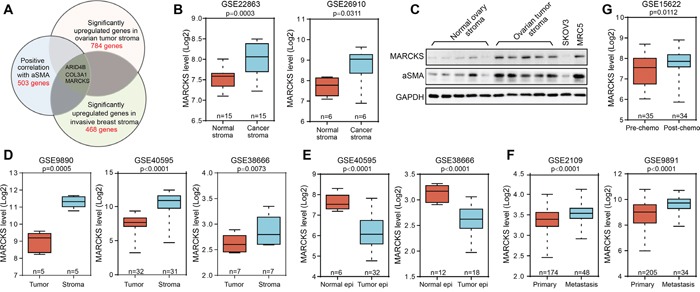
The upregulation and significance of MARCKS in ovarian tumor stroma **A.** Graphical representation of computational analysis using the microdissected stromal profiling datasets of high grade EOC (GSE40595) and invasive breast cancer (GSE9014). **B.** Normalized expression of MARCKS in microdissected tumor stroma versus matched microdissected normal stromal tissues (15 samples in GSE22863 of lung cancer and 6 samples in GSE26910 of prostate cancer). **C.** Western blot analysis of MARCKS and αSMA in normal ovary stromal tissues and high grade ovarian tumor stromal tissues. SKOV3 served as a negative and MRC5 served as a positive control for stromal molecules expression. GAPDH was used as the loading control. **D.** Comparison of MARCKS expression in microdissected tumor stromal tissues with that of microdissected tumor epithelial tissues using three EOC profiles (GSE9890, GSE40595 and GSE38666). **E.** Comparison of MARCKS expression in microdissected tumor epithelial tissues with that of microdissected normal epithelial tissues using two EOC profiles (GSE40595 and GSE38666). **F.** Normalized expression of MARCKS in primary tumors and the metastases using two EOC profiling data (GSE2109 and GSE9891). **G.** Normalized expression of MARCKS in EOC tumor samples obtained before and after chemo-intervention using the GSE15622 dataset.

To explore the regulatory molecules that drive gene expression representative of CAF features in EOC, we carried out an integrative analysis to identify genes that are important for CAF activation and specifically upregulated in tumor stroma. Currently, the most definitive molecular marker of CAFs is αSMA, which indicates activation of normal fibroblasts and plays a critical role in mediating communication between the stromal cells and the matrix [26, 31]. Here, we identified a cluster of 503 genes (Supplementary Table S1) that were notably positively correlated with αSMA expression in dataset GSE40595 that includes microdissected ovarian profiling data, of which fibroblasts were shown to be the major constituent [32]. By analyzing overlapping genes with the calculated 784 genes (Supplementary Table S2) in ovarian tumor stroma and 468 genes (Supplementary Table S3) in breast tumor stroma that significantly upregulated compared with their normal fibroblast counterparts in GSE40595 and breast stromal profile GSE9014, we identified ARID4B, COL3A1 and MARCKS as candidate targets for controlling stromal activation (Figure 1A). Among the genes identified, ARID4B expression was not correlated with EOC patient survival (Supplementary Figure 1A and 1B). In contrast, higher COL3A1 expression was notably correlated with worse patient outcome (Supplementary Figure 1C and 1D). However, the role of collagen family members in remodeling ECM were well studied [33], and they were more likely to be the downstream functional executors than the upstream regulators of stromal activation. MARCKS was selected for further study due to its reported role in regulating tumor cell adhesion and migration [34–36]; its impact on CAFs activity has not been well studied. The advantageous expression of MARCKS in cancer stroma was further confirmed in another two stoma profiling datasets of lung and prostate cancer and in tumor stroma of EOC patient samples (Figure 1B and 1C). Compared with its expression in the tumor epithelial compartment, MARCKS was noted specifically expressed in the stromal compartment as determined by analysis of EOC-related datasets (Figure 1D). In contrast, MARCKS level was reduced in the tumor epithelial cells compared with normal ovary epithelial tissues (Figure 1E). Data mining in EOC profiling data also showed that MARCKS expression was elevated along with disease metastasis (Figure 1F) or after chemo-intervention (Figure 1G). All these data demonstrated that MARCKS was highly expressed in ovarian stroma and might regulate the stromal CAF activity.

### MARCKS is specifically expressed in stromal CAFs and correlates with patient outcome

**Figure 2:**
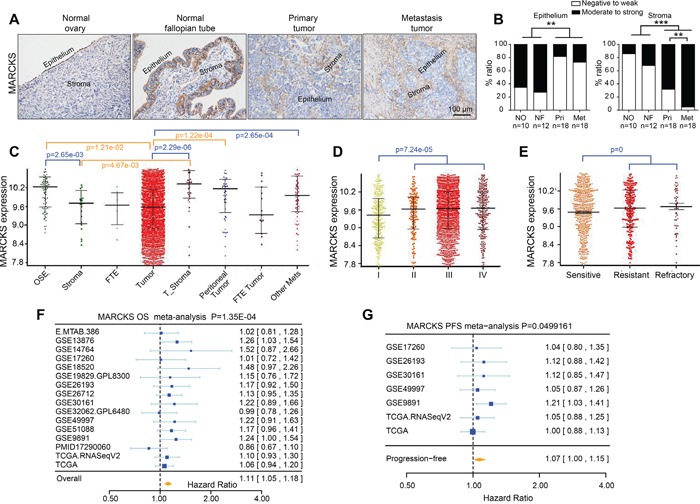
Immunohistochemical staining of MARCKS in ovarian tissues and its clinical relevance **A.** Immunohistochemistry (IHC) detection of MARCKS in a series of ovarian tissues including normal ovaries, normal fallopian tubes, paired primary and metastatic tumor tissues of high grade EOC patients. **B.** Scoring of MARCKS in the epithelial and stromal compartment of the above IHC stained tissues. Gene expression profiles of MARCKS in ovarian cancer patients according to disease state **C.** FIGO stage **D.** clinical response **E.** in the CSIOVDB dataset of ovarian cancer [37]. Abbreviation: OSE, ovarian surface epithelium; FTE, fallopian tube epithelium; Mets, metastasis. Meta-analysis depicting the forest plot of MARCKS expression as a univariate predictor of overall survival (OS) **F.** and progression-free survival (PFS) **G.,** using several datasets with applicable genes expression and survival information of high grade EOC patients. (**P<0.01; ***P<0.001).

To further validate the expression pattern of MARCKS during OC progression, we analyzed MARCKS expression in tissue samples including 10 normal ovarian tissues, 12 normal fallopian tube tissues and 18 pairs of primary and metastatic EOC tissues. Immunohistochemistry (IHC) demonstrated that 65% of normal ovary and 72% of the fallopian tube tissue versus 18% of the primary and 27% of the metastatic tumor samples showed moderate to strong epithelial MARCKS immunostaining, whereas 68% of the primary and 95% of the metastatic tumor sample versus 14% of the normal ovary and 32% of the fallopian tube tissues showed moderate to strong stromal MARCKS immunostaining. (Figure 2A and 2B). Additionally, integrative analysis of MARCKS was conducted in the CSIOVDB dataset, which includes transcriptomic profiles of 3,431 ovarian cancer specimens [37]. We found that MARCKS expression was elevated in tumor stroma and reduced in tumor epithelia as OC progresses (Figure 2C), and was increased in patients with a higher FIGO stage (Figure 2D) or in those that developed chemoresistance or disease recurrence (Figure 2E).

Next, we assessed the effect of tumoral MARCKS expression on patient survival. Firstly, we analyzed its significance in a large set of EOC samples included in curatedOvarianData [38], which includes expression data as well as survival information. MARCKS mRNA expression was found to be significantly correlated with poor patient overall survival (OS) (HR=1.11, *p*=1.35e-4) (Figure 2F), and with worse progression-free survival (PFS) in EOC patients (HR=1.07, *p*=0.0499) (Figure 2G). The impact of MARCKS on patients survival was further analyzed in a large annotated database of breast and lung cancer patient samples. Kaplan–Meier analyses showed that elevated MARCKS was significantly correlated with decreased OS (HR=1.28, p=0.039), post-progression survival (PPS) (HR=1.63, p=0.00066) and relapse-free survival (RFS) (HR=1.59, p=2.1e-14) (Supplementary Figure 2A) in breast cancer and with decreased OS (HR=1.3, p=5.7e-05), PPS (HR=1.46, p=0.008) and first progression (FP) (HR=1.95, p=1.5e-10) (Supplementary Figure 2B) in lung cancer. These findings indicated that MARCKS was primarily restricted to stromal CAFs and it served as an independent prognostic factor of poor outcome in various cancers.

### MARCKS facilitates proliferation, chemotherapeutical resistance and migration of CAFs

**Figure 3:**
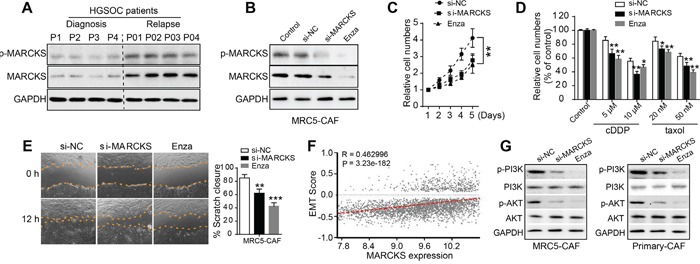
MARCKS is involved in CAF proliferation, chemotherapeutical resistance and migration **A.** Western blot analysis of MARCKS and p-MARCKS in purified CAFs from EOC tumor tissues obtained either at initial diagnosis or at disease recurrence after several cycles of chemotherapeutical intervention. **B.** Immunoblotting of MARCKS and p-MARCKS protein expression in MRC5-CAF cells in the absence or presence of si-NC or si-MARCKS or enzastaurin (Enza, 1 μM) treatment for 48 h. **C.** CCK8 assay detecting relative cell numbers of MRC5-CAFs in the si-NC or si-MARCKS or Enza (1 μM) intervention groups for various duration (1-5 days). **D.** Detection of relative cell viability in the si-NC or si-MARCKS or Enza (1 μM) pretreated groups of MRC5-CAFs, in the absence or presence of cDDP or taxol intervention at various dosage for 48 h. **E.** Representative images demonstrating and statistical analysis of wound closure capacity of MRC5-CAFs in si-NC or si-MARCKS or Enza (1 μM) treatment groups at 0 h and 12 h time points. **F.** Spearman correlation analysis of MARCKS expression with that of normalized EMT score in integrated large cohorts of ovarian cancer [38]. **G.** Western blot analysis of PI3K, p-PI3K, AKT and p-AKT expression in MRC5-CAFs and primary ovarian CAFs, in the presence of si-NC or si-MARCKS or Enza (1 μM) treatment for 72 h. GAPDH served as the loading control. Data are expressed as mean ± s.e.m. (*P < 0.05; **P < 0.01).

In accordance with the above finding that MARCKS was elevated after chemotherapeutical intervention, we performed immunoblotting in stromal fibroblasts isolated from OC tumor tissues before and after chemo-treatment and found that MARCKS protein was drastically induced by cytotoxic agents (Figure 3A). Given the increase in MARCKS expression as OC progresses or under chemo-intervention, we hypothesized a potential role of MARCKS in OC metastasis and disease recurrence by activating tumor stroma. To explore the impact of MARCKS on CAF features, we used MARCKS-specific small interfering RNA (siRNA) and the PKC inhibitor enzastaurin (Enza) to attenuate MARCKS in MRC5 fibroblast cell line-induced CAFs (MRC5-CAFs). Western blot confirmed that MARCKS and p-MARCKS proteins were significantly suppressed with MARCKS siRNA or Enza intervention (Figure 3B). Cell viability assays revealed that CAF proliferation was notably attenuated after MARCKS inhibition (Figure 3C). In addition, CAF cells were much more sensitive to cytotoxic agents such as cisplatin (cDDP) or taxol (Figure 3D). Similarly, CAF cells showed a diminished migratory capacity after silencing of MARCKS (Figure 3E). As the developmental program epithelial-mesenchymal transition (EMT) correlates with malignant cells migration and chemosensitivity [39], we analyzed MARCKS expression with EMT associated signature genes and found a strong positive relationship between them in large profiling cohorts of ovarian cancer (Figure 3F). These preliminary observations suggested that MARCKS maintained the migratory and proliferative capacity and mediated chemoresistance of CAFs. Moreover, MARCKS has been reported to be associated with the PI3K/AKT pathway [40], which is supposed to be a classical regulator of malignant behaviors in various cancer cells. We therefore performed immunoblotting to detect the influence of MARCKS expression on PI3K/AKT signaling in fibroblast cells and found that MARCKS inhibition remarkably attenuated the PI3K/AKT cascade in MRC5-CAFs and primary ovarian CAFs (Figure 3G).

### MARCKS contributes to perpetual activation of stromal fibroblasts

**Figure 4:**
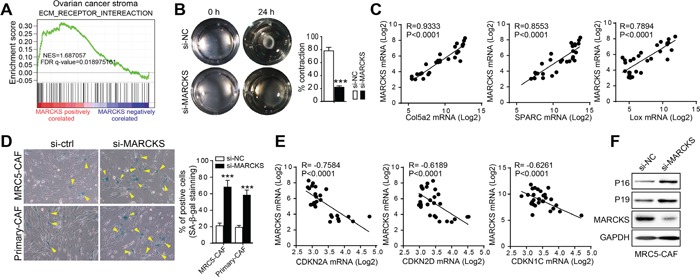
MARCKS overexpression contributes to the constitutive activation of stromal fibroblasts **A.** “ECM-RECEPTOR-INTEREACTION” GSEA plot of enrichment of gene expression in the genesets positively correlated with MARCKS expression in ovarian stromal profile of GSE40595 dataset. **B.** Representative images demonstrating and statistical analysis of collagen gel contraction capacity of MRC5-CAFs transfected with si-NC or si-MARCKS at 0 h and 24 h time points. **C.** Pearson correlation analysis of ECM molecules as Col5a2, SPARC and Lox with that of MARCKS expression in 39 microdissected ovarian stromal profiles of GSE40595 dataset. **D.** Representative images and statistical analysis of β-galactosidase staining in MRC5-CAFs and primary ovarian CAFs, after treatment with si-NC or si-MARCKS for 72 h. **E.** Pearson correlation analysis of senescence related molecules as CDKN2A, CDKN2D and CDKN1C with that of MARCKS expression in 39 microdissected ovarian stromal profiles of the GSE40595 dataset. **F.** Western blot analysis of the above senescence associated molecules as P16 and P19 in MRC5-CAF cells after 72 h treatment with si-NC or si-MARCKS. GAPDH served as the loading control. Data are expressed as mean ± s.e.m. (***P < 0.001).

In GSE40595, we performed gene set enrichment analysis (GSEA) in 31 microdissected ovarian tumor stromal samples and identified that the “ECM-RECEPTOR-INTEREACTION” as a significantly enriched signature among the gene sets positively correlated with MARCKS expression (NES=1.69, FDR q=0.019) (Figure 4A). Constant remodeling of the ECM is the fundamental role of activated CAFs [41]. Indeed, collagen contraction assays revealed that MARCKS inhibition hampered the ECM contraction exerted by CAFs (Figure 4B). Meanwhile, Pearson's correlation analysis revealed that expression of key ECM remodeling molecules, such as Col5a2 (R=0.9333, *p*<0.0001), SPARC (R=0.8553, *p*<0.0001) and Lox (R=0.7894, *p*<0.0001), was significantly positively correlated with that of MARCKS in GSE40595 dataset (Figure 4C).

In addition to the ECM-remodeling characteristic, one pivotal property that distinguishes CAFs from normal fibroblasts is their constitutive activation [42], suggesting that cellular senescence is an obstacle of CAF activation. We therefore tested the effect of MARCKS inhibition on cellular senescence and showed that SA staining was remarkably enhanced in MARCKS-silenced CAFs (Figure 4D). Moreover, the expression of CDKN2A (R= −0.7584, *p*<0.0001), CDKN2D (R= −0.6189, *p*<0.0001) and CDKN1C (R= −0.6261, *p*<0.0001), representative inducers of senescence, was notably inversely correlated with that of MARCKS in GSE40595 dataset (Figure 4E). Moreover, the protein level of the aforementioned senescence-related genes, such as CDKN2A (P16) and CDKN2D (P19), was upregulated after MARCKS silencing (Figure 4F). In aggregate, these results suggested that MARCKS sustained the constitutive activation of stromal fibroblasts by constant remodeling of the ECM and suppression of cellular senescence.

### Twist1 was involved in the regulation of MARCKS on CAF activity

**Figure 5:**
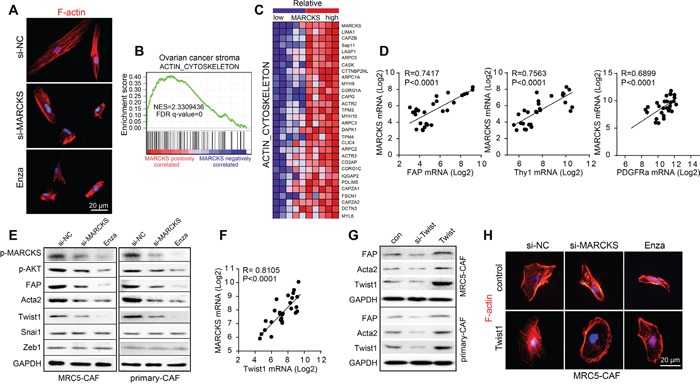
Twist1 is involved in the regulation of MARCKS on CAF activity **A.** Representative images of F-actin staining in MRC5-CAF cells after treatment with si-NC or si-MARCKS or Enza (1 μM) for 72 h. **B.** “ACTIN_CYTOSKELETON” GSEA plot of enrichment of gene expression in the genesets positively correlated with MARCKS expression in ovarian stromal profiles of GSE40595 dataset. **C.** Heat map of the top 30 genes included in the “ACTIN_CYTOSKELETON” signature expressed in the top 5 and the bottom 5 ovarian stroma samples ranked by MARCKS expression levels of GSE40595. Blue and red in the heatmap indicate genes expression that were relatively low or high, respectively. **D.** Pearson correlation analysis of CAF features markers as FAP, Thy1 and PDGFRA with that of MARCKS expression in 39 microdissected ovarian stromal profiles of GSE40595 dataset. **E.** Western blot analysis of p-MARCKS, p-AKT, FAP, Acta2, Twist1, Snai1 and Zeb1 in MRC5-CAFs and primary ovarian CAFs after 72 h treatment with si-NC or si-MARCKS or Enza (1 μM). GAPDH served as the loading control. **F.** Pearson correlation analysis of Twist1 with that of MARCKS in 39 microdissected ovarian stromal profiles of GSE40595 dataset. **G.** Western blot analysis of FAP, Acta2 and Twist1 in MRC5-CAFs and primary ovarian CAFs after 72 h treatment with si-Twist1 or Twist1 expressing plasmid. **H.** Representative images of cellular cytoskeleton by F-actin staining of MRC5-CAF cells in the treatment group of si-NC or si-MARCKS or Enza (1 μM), in the absence or presence of Twist1 expressing plasmid.

Next, we investigated the molecular mechanisms of MARCKS that regulate CAF activity in addition to its remodeling of the ECM and suppression of cellular senescence. Because MARCKS was known as an actin-binding protein whose dysregulation could alter the cellular cytoskeleton and further motility and invasion, we examined the impact of MARCKS inhibition on the CAF cytoskeleton. F-actin staining displayed that MARCKS silencing resulted in transition of cellular appearance from spread spindle architecture to a condensed appearance in MRC5-CAFs (Figure 5A). In contrast, primary normal ovarian fibroblasts presented an alignment of the actin cytoskeleton after ectopically expressing of MARCKS mediated by adenovirus (Supplementary Figure 3A). Notably, GSEA analysis revealed a strong positive correlation between genes positively related with MARCKS expression and a gene signature termed “ACTIN_CYTOSKELETON” (NES=2.33, FDR q=0) in the GSE40595 profile (Figure 5B). Furthermore, the top 30 genes included in the “ACTIN_CYTOSKELETON” signature were listed in samples with the top five and bottom five MARCKS level (Figure 5C).

To further explore the link between MARCKS and the CAF phenotype, we found that there was a statistically significant correlation between MARCKS and the pivotal CAF markers FAP (R=0.7417, *p*<0.0001), Thy1 (R=0.7563, *p*<0.0001) and PDGFRA (R=0.6899, *p*<0.0001) in GSE40595 profiles (Figure 5D). In addition, the EMT transcriptional factor Twist1, which acts downstream of AKT signaling, was reported to be capable of transforming normal fibroblasts into CAFs [43]. Therefore, we hypothesized that there might be a relationship between MARCKS and AKT/Twist1 signaling, which could potentially contribute to MARCKS regulation of CAF activation. Our results showed that AKT signaling, as well as expression of CAF markers Acta2 and FAP, was reduced dramatically after MARCKS inhibition. We also observed a specific reduction of Twist1, but there was no alteration of other EMT inducers, such as ZEB1 and Snai1, after MARCKS silencing (Figure 5E). In parallel, MARCKS overexpression activated the AKT signaling, increased Acta2 and FAP, as well as Twist1 expression in normal ovarian fibroblasts, while all the above effects exerted by MARCKS acquisition was attenuated in the presence of the PI3K/AKT inhibitor LY294002 (Supplementary Figure 3B). Moreover, Twist1 was statistically positively correlated with MARCKS in the GSE40595 dataset (R=0.8105, *p*<0.0001) (Figure 5F). Furthermore, Twist1 expression perturbance was found to alter CAF functional gene expression in MRC5-CAFs and primary ovarian CAFs (Figure 5G). Finally, overexpression of Twist1 in MARCKS-depleted CAFs led them to re-acquire the activated typical spindle appearance (Figure 5H). These findings indicated that MARCKS functions in AKT/Twist1 signaling to arrange the fibroblast cytoskeleton and the CAF marker proteins.

### Inhibition of MARCKS attenuates the supportive role of CAFs to tumor cells

**Figure 6:**
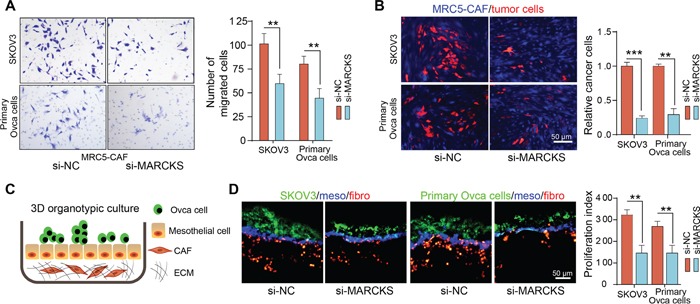
Inhibition of MARCKS attenuates the supportive role of CAFs to tumor cells **A.** Representative images and statistical analysis of cellular migration of SKOV3 and primary ovarian cancer cells, in coculture invasion system with MRC5-CAFs transfected with si-NC or si-MARCKS. **B.** Representative fluorescent images and statistical analysis of the relative cell numbers of PKH-26 (red) labeled SKOV3 and primary ovarian cancer cells, after 72 h contact coculture on top of the CMAC (blue) labeled MRC5-CAFs transfected with si-NC or si-MARCKS. **C.** Schematic depicting of the 3D organotypic culture assay with matrigel ECM substrate, fluorescently labeled CAFs (red, PKH-26), HMrSV5 mesothelial cells (blue, CMAC) and tumor cells (green, PKH-67) from bottom to the top layer. **D.** Representative fluorescent images and quantification of the proliferation index of PKH-67 labeled SKOV3 and primary ovarian cancer cells, in co-culture model as described above with variant MRC5-CAFs transfected with si-NC or si-MARCKS. Data are expressed as mean ± s.e.m. of at least three independent experiments. (**P<0.01; ***P<0.001).

We further investigated whether MARCKS activation in fibroblasts facilitates their tumor promoting function. Firstly, in a co-culture system, we found that CAFs had an attenuated attractive effect in inducing SKOV3 and primary ovarian cancer cell migration after MARCKS knock down (Figure 6A). Secondly, we plated fluorescently labeled OC cells onto feeder layers of control or MARCKS-silenced MRC5-CAFs and found a notably higher number of cancer cells in co-cultures with control CAFs than in the MARCKS-repressed group (Figure 6B). Finally, we developed a 3D organotypic co-culture model, including ECM Matrigel substrate, CAFs, and mesothelial and tumor cells labeled discriminately from bottom to the top layer, which faithfully represents the histologic and biologic microenvironment of ovarian cancer peritoneal metastasis, to evaluate the effects of MARCKS inhibition in CAF effects on tumor cells adhesion and invasion (Figure 6C). Our results demonstrated that repression of MARCKS in CAFs impaired their ability to support the growth of cocultured ovarian cancer cells. (Figure 6D). Thus, in stromal fibroblasts, MARCKS activation was necessary for the maintenance of their supportive role in facilitating tumor cell growth and invasion.

### Loss of MARCKS in fibroblasts reduces ovarian xenograft tumor growth

**Figure 7:**
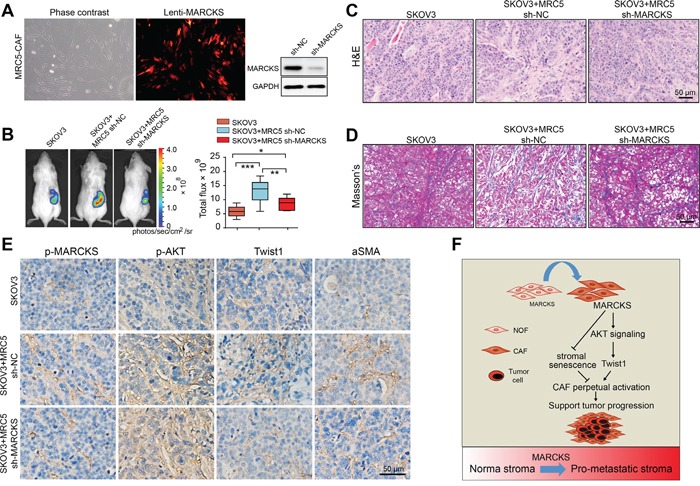
Loss of MARCKS in fibroblasts reduces its supporting of ovarian xenograft tumor growth **A.** Stably transfected MRC5-CAF cells were examined under phase-contrast microscopy (left panel) and fluorescent microscopy (right panel) to verify the >90% efficiency of lentiviral transfection. Western blot analysis confirmed the effect of MARCKS targeting lentivirus in repressing MARCKS expression. **B.** Representative bioluminescence images of mice (n=8 each group) bearing SKOV3-Luc cells alone, or co-injection with MRC5-CAFs stably transfected with either sh-NC or sh-MARCKS lentivirus at 4 weeks after tumor implantation. Bar graph showing the quantification of normalized total photon counts of the subcutaneous xenografts in mice of each group. **C.** H&E and **D.** Masson trichrome staining and **E.** Immunohistochemical staining of p-MARCKS, p-AKT, Twist1 and aSMA in tumor sections from mice in SKOV3-Luc cells solitary group, and in groups coinjected with sh-NC or sh-MARCKS lentivirus transduced MRC5-CAFs. **F.** A graphical illustration of the molecular signaling events involved in MARCKS regulation of CAF activation. MARCKS overexpression suppresses cellular senescence and boosts the activation of AKT/Twist1 signaling to maintain the CAF features, thus supporting tumor cells growth and invasion. (*P<0.05; **P<0.01; ***P<0.001).

To further explore the importance of MARCKS in stromal fibroblasts supporting of tumor growth *in vivo*, lentiviruses containing the scrambled sequence (NC) or MARCKS-targeting sequence were obtained and stably transduced into MRC5-CAFs. High and stable transduction efficiency (>90%) of MARCKS was confirmed by the red fluorescent signal from the Lenti–vector and immunoblotting (Figure 7A). We mixed MRC5-CAFs stably transfected with either sh-NC or sh-MARCKS with the SKOV3-Luc cancer cells and coinjected them subcutaneously into NOD/SCID mice. Tumors arising from SKOV3-Luc cells co-injected with sh-NC CAFs grew significantly faster than those co-injected with sh-MARCKS CAFs, both of which groups developed larger tumors than the SKOV3-Luc only group (Figure 7B). To better understand these results, we excised tumors at the end of the experiment and examined the tumor tissue histology. Both H&E and Masson trichrome staining revealed that the stroma-rich degree of tumors from mice coinjected with SKOV3-Luc cells and sh-MARCKS CAFs was much weaker than the sh-NC CAFs coinjection group while stronger than the SKOV3-Luc solitary group, indicating a role of MARCKS in contributing to the tumor stroma activation. (Figure 7C and 7D). Subsequently, IHC analysis of the obtained tumor sections confirmed the diminished stromal staining of MARCKS activation and AKT signaling, as well as the attenuated stromal Twist1 expression and the resultant αSMA staining, in the sh-MARCKS group compared with that of the sh-NC co-injection group. (Figure 7E). Taken together, our data showed here that the MARCKS expression supports the emergence of a CAF-like cell state, impacting tumor progression through stroma–tumor communication that alters the tumor-supporting role of CAFs (Figure 7F).

## DISCUSSION

The key finding from this study is that MARCKS acts as a potent regulator of CAF activity in EOC. Suppression of MARCKS resulted in fibroblast senescence and the loss of the CAF phenotype through the attenuation of the AKT/Twist1 cascade signaling, which contributes to the maintenance of stromal CAF activity. High MARCKS mRNA expression was significantly associated with poor survival in EOC patients. Our finding demonstrates the pivotal role of stromal MARCKS upregulation in propelling EOC advancement and suggests that MARCKS could be a target of choice in targeting the stromal constituent of EOC.

After analyzing the genesets that were specifically elevated in the microdissected stroma of both ovarian and invasive breast cancer, we found that they comprises nearly all the molecules included in the aforementioned “stromal-response” signature and “reactive stroma signature” characterizing disease outcome and chemoresistance, respectively. Therefore, our obtained activated stroma signatures could serve as reliable platform to analyze regulators of stromal CAF activation. Eventually, we discovered three candidate genes that were significantly overexpressed and closely correlated with αSMA in ovarian stroma. In contrast, in a recent report depicting six master regulators (AEBP1, HOPX, PRRX1, SNAI2, ZEB1, ZEB2) of the TCGA “mesenchymal” subtype, they employed a network-based strategy to uncover the molecular mechanism underlying the discrete mesenchymal subtype in whole tumor tissues of serous OC [44]. Comparatively, we conducted the analysis in isolated stromal tissues and used αSMA as an optimal representation of CAF traits from various origins. Among the genes identified, ARID4B was reported as a prometastatic gene [45] and served as an independent predictor of disease outcome in breast cancer, while its expression was not correlated with OC patient survival. In contrast, higher COL3A1 expression was correlated with platinum resistance [46] and worse patient outcome in OC, and it was more likely to be the downstream effector than the upstream regulator of CAF activation. To this end, MARCKS was chosen for further study of its potent role in driving stromal activation, considering its recognized role in regulating cellular cytoskeleton.

MARCKS was highly accordant with the requirements for being a supposed CAF phenotype activator, based on our observation that MARCKS sustained constitutive CAF activation and facilitated ovarian xenograft growth. The striking observation was that MARCKS was increased in the stromal compartment while decreased in the epithelial compartment along OC progression. This opposite alteration of MARCKS raised the possibility that MARCKS exerts its role particularly in the context of stromal CAFs rather than the epithelial counterpart. Consistent with previous findings that MARCKS was associated with metastatic potential in lung cancer and colon cancer cells [28, 47], we showed here that MARCKS positively regulates fibroblast proliferation and migration. Furthermore, in accordance with that MARCKS was upregulated in tumor stroma after chemo-intervention, we found that suppression of MARCKS sensitized CAFs to cytotoxic agents, supporting its role in regulating chemosensitivity. Mechanistically, it was reported in multiple myoma that MARCKS inhibition causes cell cycle arrest and apoptosis [48]. Similarly, we first showed that MARCKS suppression resulted in cellular senescence, contributing to the inactivation of the CAF features. Moreover, we found that MARCKS was positively correlated with “actin-cytoskeleton” signature genes in stromal profiles, and MARCKS inhibition led to the loss of typical alignment of the actin cytoskeleton representative of CAF phenotype. This observation was in accordance with the notion that MARCKS primarily regulates cell migration through actin cytoskeletal remodeling [49].

Constitutive activation is an essential property of CAFs that distinguishes them from normal fibroblasts [50]. In this scenario, senescence is an obstacle CAFs have to overcome to maintain their activation and propelling role in the tumor microenvironment. Accordingly, we found that MARCKS was negatively correlated with senescence-associated genes, and MARCKS inhibition caused obvious cellular senescence in CAFs, supporting its role in facilitating CAF activation. Mechanistically, the dynamic alteration of cellular actin cytoskeleton correlates with cell cycle transition as well as cellular senescence. MARCKS might exert its influence on cellular senescence through the maintaining of the alignment of actin cytoskeleton in fibroblasts. However, it has been recently reported that senescent fibroblasts can acquire a cancer-promoting senescence-associated secretory phenotype (SASP) which correlates with tumor progression under certain conditions. The exact mechanism involved in MARCKS regulation of cellular senescence and whether MARCKS inhibition contributed to the acquisition of SASP remained to be elucidated by future studies.

MARCKS inhibition was shown to suppress the AKT signaling, a pathway that controls nearly all the malignant behaviors of tumor cells [51]. Mechanistically, MARCKS carries out the actin cytoskeleton arrangement function with its polybasic effector domain that cross-links actin filaments into bundles and sequesters bisphosphate (PIP2) [40], which is a component of phosphatidylinositol 3′-kinase (PI3K)/AKT pathways. Accordingly, it was recently reported that activation of the PI3K/Akt signaling cascade in the tumor stroma drives breast tumor regression [52], emphasizing the pivotal role of AKT in CAFs as in tumor cells. In addition, a key EMT transcriptional factor, Twist1, was shown to be responsible for MARCKS action on CAF characteristics, similar to that Twist1 induced the transformation of normal fibroblasts in gastric cancer [43]. These observed effects of Twist in CAFs complement the role of Twist1 in cancer advancement. Therefore, the AKT/Twist1 signaling activated by MARCKS overexpression sustain the CAF traits in EOC. Understanding the key regulators controlling CAF activation, such as MARCKS in our work, could help in stratifying patients for individualized therapy and better predicting disease outcomes.

In summary, we demonstrated here that MARCKS contributed to constitutive CAF activation in OC, involving the suppression of cellular senescence and activation of the AKT/Twist1 signaling. Of particular importance, we uncovered that MARCKS overexpression defined a poor prognosis in OC patients. Our observations suggest that inhibition of MARCKS and the related signaling network to target stromal activation could be a potential approach in targeting the cooperative tumor stroma of OC.

## MATERIALS AND METHODS

### Cell culture

The EOC cell line SKOV3 was purchased from ATCC (Rockville, MD, USA). Fibroblast cell line MRC-5 was obtained from the cell bank of the Chinese Academy of Sciences. Mesothelial cell line HMrSV5 was obtained from Jennio Biological Technology (Guangzhou, China). All the cell lines were authenticated by their source organizations prior to purchase, routinely checked for mycoplasma contamination and used within 4 months after frozen aliquot recovery. Primary normal ovarian fibroblasts (NFs), ovarian CAFs and tumor cells were obtained from EOC patient tumor tissues following procedures as previously described [53]. SKOV3 and primary EOC cells were maintained in McCoy's 5A medium and MRC-5, HMrSV5, primary NFs and CAFs were cultured in DMEM/F-12 medium with 10 % FBS and 1% penicillin/streptomycin (Thermo Scientific), at 37°C in a 5% CO_2_ and 80% humidity incubator. TGF-β1 (50 ng/ml) (Sigma, St. Louis, MO, USA) was added to MRC5 cultures for 7-10 days to obtain the transformed CAFs (MRC5-CAFs). SKOV3 cells had been stably transduced with CMV-Fluc-IRES-RFP lentiviral particles (GeneChem, shanghai, China) and designated as SKOV3-Luc previously, which were further used in animal living imaging experiment.

### GEO data sets analysis and pearson correlation

Gene expression data (GSE40595, GSE9014, GSE22863, GSE26910, GSE9890, GSE9891, GSE38666, GSE2109, GSE15622 profiling data) were downloaded as raw signals from Gene Expression Omnibus (http://www.ncbi.nlm.nih.gov/geo), interpreted, normalized and log2 scaled using the online analysis tool GCBI website (https://www.gcbi.com.cn). Exploring of differentially expressed gene sets between normal and cancer stromal profiles in GSE40595 and GSE9014 was also performed via the GCBI online tool. Among the above normalized data sets, MARCKS probe expressions were extracted and compared in patient samples stratified by different clinical parameters. In stromal profiles of the GSE40595 dataset, MARCKS probe signals were extracted and analyzed for Pearson's correlation with ECM molecules (Col5a2, SPARC, Lox), senescence-related molecules (CDKN2A, CDKN2D, CDKN1C), CAF marker molecules (FAP, Thy1, PDGFRA) and Twist1 probes.

### Gene set enrichment analysis

To determine the enrichment of specific gene signatures in the genesets positively correlated with MARCKS expression in stromal profiling of the GSE40595 dataset, gene set enrichment analysis (GSEA) was performed using the publicly available desktop application from the Broad Institute (http://www.broad.mit.edu/gsea/software/software_index.html). Gene signatures associated with CAF features such as “ECM-RECEPTOR-INTEREACTION” and “ACTIN_CYTOSKELETON” were selected from the MSIGDB signature datasets.

### Western blot analysis and immunofluorescence

Cells were lysed with RIPA lysis buffer (Beyotime, Shanghai, China) supplemented with a protease inhibitor cocktail (Roche). Protein concentration was measured with a bicinchoninic acid (BCA) assay (Thermo Scientific), and 40 μg of total lysate for each sample was subjected to SDS-PAGE followed by blotting with the indicated primary antibodies. Antibodies for MARCKS (#5607), p-MARCKS (#8722), PI3K (#3811), p-PI3K (#3821), AKT (#2920), p-AKT (#11962) were purchased from Cell Signaling Technology (Beverly, MA, USA). Antibodies against P16 (EPR1473), P19 (ab26911), αSMA (ab7817) and GAPDH (ab9485) were obtained from Abcam Biotechnology (Abcam, CA, USA). Then incubated with the corresponding HRP-linked secondary antibody (Abcam) and finally detected using an enhanced ECL system (Pierce). Immunofluorescence of the cytoskeleton was performed as conducted previously [54]. F-actin was visualized in cells by incubation with rhodamine-conjugated phalloidin (Thermo Scientific). Fluorescence images were taken using an Olympus BX53 microscope (Olympus, Tokyo, Japan).

### Immunohistochemistry and masson's trichrome staining

Human tissues were obtained from the Department of Gynecology of Tongji Hospital (Wuhan, China). Informed consent was obtained from all patients. EOC tumor tissues including matched primary tumors and metastases were from patients diagnosed with advanced (stages III and IV) serous adenocarcinoma. Normal ovaries and fallopian tubes were obtained from patients who underwent prophylactic adnexectomy due to benign uterine lesions. Immunohistochemical staining for MARCKS, p-MARCKS, p-AKT, Twist1 and αSMA expression was conducted as described in our prior study [55]. Immunostaining scoring was evaluated on the basis of staining intensity and positively stained areas by three independent observers as previously described [56]. These data were analyzed as a continuum and differences between groups were compared with a semiquantitative method. Masson's trichrome (Sigma, HT15) staining was performed as described previously [57] on paraffin embedded sections of xenograft tumors.

### Survival analysis

To evaluate the influence of MARCKS expression on disease outcome of EOC patients, we performed a meta-analysis of 2970 EOC patient expression profiles and generated forest plot using the ‘curatedOvarianData’ Bioconductor package [38]. Survival curves were calculated using the Kaplan–Meier method, conducted with the R Bioconductor ‘survival’ package. Kaplan–Meier curves were generated using a database of public microarray data sets (http://kmplot.com) via website interface 2015. MARCKS probe (201669_s_at), probe (201669_s_at) and probe (201670_s_at) were selected for calculating OS, PPS and RFS in breast cancer patients, respectively [58], and probe (213002_s_at), probe (201670_s_at) and probe (201668_s_at) was selected for generating Kaplan–Meier plots calculating the OS, PPS and FP in lung cancer patients, respectively [59]. The parameters were split patients by median and auto-select best cut-off, and all other parameters were defaults.

### Cell viability assay

For cell viability assays, 6,000 MRC5-CAF cells, tranfected with either si-NC or si-MARCKS or treated with enzastaurin (1 μM) (Selleck), were seeded in 96-well plates. After plating, cells were subjected to various treatments for the indicated times. Cell viability was assayed with the Cell Counting Kit-8 (Dojindo Laboratories, Kumamoto, Japan) according to the manufacturer's instructions. Absorbance at 450 nm (OD450) was determined for each well using an automated microplate reader (SpectraMax 190; Molecular Devices, USA). Relative cell number was presented as the absorbance value compared with that of control cells. Each assay was performed in triplicate.

### Transfection of siRNA, plasmid, lentivirus and adenovirus

MRC5-CAFs or primary CAFs were transfected with Lipofectamine 2000 reagent (Invitrogen) using either negative control (si-NC), MARCKS specific (si-MARCKS) or Twist1 specific siRNAs (Santa Cruz Biotechnology, CA, USA) according to the manufacturer's protocol. X-tremeGENE HP DNA Transfection Reagent (Roche) was used for plasmid transfection of the Twist1-expressing plasmid PTK-Twist (#36977) obtained from Addgene (Cambridge, MA, USA) according to the manufacturer's protocol. Lentiviral shRNA targeting MARCKS was obtained from Genechem (Shanghai, China) for knocking down of MARCKS in MRC5-CAFs. GFP-based flow cytometry sorting was performed to select cells stably transduced with lentivirus targeting MARCKS (Lenti-MARCKS) or control vector (Lenti-vector). Recombinant adenovirus expressing MARCKS was obtained from Genechem for transient overexpression of MARCKS in primary NFs.

### Collagen gel contraction assay

A total of 4×10^5^ MRC5-CAFs transfected either with si-NC or si-MARCKS were suspended in a collagen gel mixture composed of 100 μL of collagen mix (68.75 μl DMEM/F-12 medium, 0.72 μl 1N NaOH, 31.25 μl Rat Tail Collagen, Type 1) (Thermo) and 100 μL DMEM/F-12 medium per well in a 24-well ultra-low attachment plate (Corning Life Sciences, Corning, NY). Gels were photographed, and the area was measured using ImageJ software and expressed as a percentage of the original well area. All contraction assays were performed in triple.

### β-gal staining

SA-β-gal activity was measured using a β-gal staining kit (Cell Signaling) according to the manufacturer's instructions. 72 h after transfection of either si-NC or si-MARCKS in MRC5-CAFs or primary CAFs in 6-well plates, cells were fixed for 15 min with 1× fixation solution and incubated overnight at 37°C with 1× staining solution mix. Blue staining was observed and counted under a microscope.

### Scratch assay

MRC5-CAFs transfected with either si-NC or si-MARCKS or treated with enzastaurin (1 μM) (Selleck) were seeded in 6-well plates. After reaching confluency, monolayers were scratched with a sterile pipette tip to make a scratch of approximately 0.4–0.5 mm in width and cells were cultured in serum deprived medium. Scratch closure was photographed, and the area was measured using ImageJ software and expressed as a percentage of the original area. All scratch assays were performed in triplicae.

### Coculture invasion assay

MRC5-CAFs (1 × 10^5^) transfected with si-NC or si-MARCKS were seeded in 24-well plates and allowed to attach for 12 h. Then, 2 × 10^4^ SKOV3 cells or 3 × 10^4^ primary EOC cells were added to the Boyden chambers (8 μm pore size; Corning Life Sciences). After incubation for 24 h, the nonmotile cells were removed with a cotton swab. The remaining cells at the lower surface of the filter were fixed with cold methanol and stained with 0.1% (w/v) crystal violet (Sigma). The number of migrating cells in each chamber was counted in five randomly chosen fields under microscope for three independent experiments.

### 2D contact co-culture and 3D organotypic culture

For 2D supportive coculture, MRC5-CAFs labeled with CMAC (blue, Thermo) transfected either with si-NC or si-MARCKS were plated at near confluency. 24 h later, cancer cells labeled with PKH-26 (Read, Sigma) were seeded on top of the CAFs (1:6 ratio of cancer cell : CAFs) and allowed to grow for 72 h. Flow cytometry was used to quantify the tumor cell amounts. Three-dimentional organotypic culture was performed as described previously by Ernst Lengyel [60]. Fluorescently labeled MRC5-CAFs (PKH-26) transfected with either si-NC or si-MARCKS, mesothelial cells (CMAC) and tumor cells (green, PKH-26, sigma) were added from bottom to the top layer. After 10 d of tumor cells implantation, organotypic gels were harvested and embedded in OCT. Fluorescent images of frozen sections were taken under an Olympus BX53 microscope (Olympus). Quantification of the invasion assays was performed as described previously [51] using ImageProPlus software.

### Animal assay

The animal study was performed with the approval of the Committee on the Ethics of Animal Experiments in the Hubei province. 4-6-week-old female NOD/SCID mice were housed and maintained in laminar flow cabinets under specific pathogen-free condition. SKOV3-Luc (2 × 10^6^) cells were inoculated subcutaneously in right back of the mice, either alone or co-injected with 3 × 10^6^ sh-NC CAFs or with sh-MARCKS CAFS (n=8 per group). Tumor growth was monitored by caliper measurements twice weekly. Approximately 4 weeks later, mice were anaesthetized with 1% pentobarbital sodium, and imaged with the IVIS SPECTRUM system (Caliper, Xenogen, USA). Total flux (photons/s) of xenografts was analyzed using Living Image version 4.3.1 software. Tumor xenografts in each group were collected at the end point for further immunohistochemical study.

### Statistics

Data are presented as the mean value ± s.e.m from at least three independent experiments. Statistical analyses were performed with Prism 6.0 GraphPad software. Single comparisons between two groups were determined by Student's t-test. Comparisons between multiple groups were determined by one-way ANOVA followed by Tukey post-test. For correlation studies, statistical significance was calculated by Pearson's correlation analysis. P values < 0.05 were considered significant.

## SUPPLEMENTARY FIGURES AND TABLES





## References

[1] Bowtell DD (2010). The genesis and evolution of high-grade serous ovarian cancer. Nature reviews Cancer.

[2] Vang R, Shih Ie M, Kurman RJ (2009). Ovarian low-grade and high-grade serous carcinoma: pathogenesis, clinicopathologic and molecular biologic features, and diagnostic problems. Advances in anatomic pathology.

[3] Shih Ie M, Kurman RJ (2004). Ovarian tumorigenesis: a proposed model based on morphological and molecular genetic analysis. The American journal of pathology.

[4] Tothill RW, Tinker AV, George J, Brown R, Fox SB, Lade S, Johnson DS, Trivett MK, Etemadmoghadam D, Locandro B, Traficante N, Fereday S, Hung JA, Chiew YE, Haviv I, Gertig D (2008). Novel molecular subtypes of serous and endometrioid ovarian cancer linked to clinical outcome. Clinical cancer research.

[5] (2011). Integrated genomic analyses of ovarian carcinoma. Nature.

[6] Konecny GE, Wang C, Hamidi H, Winterhoff B, Kalli KR, Dering J, Ginther C, Chen HW, Dowdy S, Cliby W, Gostout B, Podratz KC, Keeney G, Wang HJ, Hartmann LC, Slamon DJ (2014). Prognostic and therapeutic relevance of molecular subtypes in high-grade serous ovarian cancer. Journal of the National Cancer Institute.

[7] Verhaak RG, Tamayo P, Yang JY, Hubbard D, Zhang H, Creighton CJ, Fereday S, Lawrence M, Carter SL, Mermel CH, Kostic AD, Etemadmoghadam D, Saksena G, Cibulskis K, Duraisamy S, Levanon K (2013). Prognostically relevant gene signatures of high-grade serous ovarian carcinoma. The Journal of clinical investigation.

[8] Bookman MA, Gilks CB, Kohn EC, Kaplan KO, Huntsman D, Aghajanian C, Birrer MJ, Ledermann JA, Oza AM, Swenerton KD (2014). Better therapeutic trials in ovarian cancer. Journal of the National Cancer Institute.

[9] Coleman RL, Monk BJ, Sood AK, Herzog TJ (2013). Latest research and treatment of advanced-stage epithelial ovarian cancer. Nature reviews Clinical oncology.

[10] Waldron L, Haibe-Kains B, Culhane AC, Riester M, Ding J, Wang XV, Ahmadifar M, Tyekucheva S, Bernau C, Risch T, Ganzfried BF, Huttenhower C, Birrer M, Parmigiani G (2014). Comparative meta-analysis of prognostic gene signatures for late-stage ovarian cancer. Journal of the National Cancer Institute.

[11] Kalluri R, Zeisberg M (2006). Fibroblasts in cancer. Nature reviews Cancer.

[12] Giannoni E, Bianchini F, Masieri L, Serni S, Torre E, Calorini L, Chiarugi P (2010). Reciprocal activation of prostate cancer cells and cancer-associated fibroblasts stimulates epithelial-mesenchymal transition and cancer stemness. Cancer research.

[13] Drake LE, Macleod KF (2014). Tumour suppressor gene function in carcinoma-associated fibroblasts: from tumour cells via EMT and back again?. The Journal of pathology.

[14] Lau TS, Chung TK, Cheung TH, Chan LK, Cheung LW, Yim SF, Siu NS, Lo KW, Yu MM, Kulbe H, Balkwill FR, Kwong J (2014). Cancer cell-derived lymphotoxin mediates reciprocal tumour-stromal interactions in human ovarian cancer by inducing CXCL11 in fibroblasts. The Journal of pathology.

[15] Xing F, Saidou J, Watabe K (2010). Cancer associated fibroblasts (CAFs) in tumor microenvironment. Frontiers in bioscience (Landmark edition).

[16] Ostman A, Augsten M (2009). Cancer-associated fibroblasts and tumor growth-bystanders turning into key players. Current opinion in genetics & development.

[17] Alkasalias T, Flaberg E, Kashuba V, Alexeyenko A, Pavlova T, Savchenko A, Szekely L, Klein G, Guven H (2014). Inhibition of tumor cell proliferation and motility by fibroblasts is both contact and soluble factor dependent. Proceedings of the National Academy of Sciences of the United States of America.

[18] Yeung TL, Leung CS, Wong KK, Samimi G, Thompson MS, Liu J, Zaid TM, Ghosh S, Birrer MJ, Mok SC (2013). TGF-beta modulates ovarian cancer invasion by upregulating CAF-derived versican in the tumor microenvironment. Cancer research.

[19] Leung CS, Yeung TL, Yip KP, Pradeep S, Balasubramanian L, Liu J, Wong KK, Mangala LS, Armaiz-Pena GN, Lopez-Berestein G, Sood AK, Birrer MJ, Mok SC (2014). Calcium-dependent FAK/CREB/TNNC1 signalling mediates the effect of stromal MFAP5 on ovarian cancer metastatic potential. Nature communications.

[20] Qiu W, Hu M, Sridhar A, Opeskin K, Fox S, Shipitsin M, Trivett M, Thompson ER, Ramakrishna M, Gorringe KL, Polyak K, Haviv I, Campbell IG (2008). No evidence of clonal somatic genetic alterations in cancer-associated fibroblasts from human breast and ovarian carcinomas. Nature genetics.

[21] Busuttil RA, George J, Tothill RW, Ioculano K, Kowalczyk A, Mitchell C, Lade S, Tan P, Haviv I, Boussioutas A (2014). A signature predicting poor prognosis in gastric and ovarian cancer represents a coordinated macrophage and stromal response. Clinical cancer research.

[22] Ryner L, Guan Y, Firestein R, Xiao Y, Choi Y, Rabe C, Lu S, Fuentes E, Huw LY, Lackner MR, Fu L, Amler LC, Bais C, Wang Y (2015). Upregulation of Periostin and Reactive Stroma Is Associated with Primary Chemoresistance and Predicts Clinical Outcomes in Epithelial Ovarian Cancer. Clinical cancer research.

[23] Navab R, Strumpf D, Bandarchi B, Zhu CQ, Pintilie M, Ramnarine VR, Ibrahimov E, Radulovich N, Leung L, Barczyk M, Panchal D, To C, Yun JJ, Der S, Shepherd FA, Jurisica I (2011). Prognostic gene-expression signature of carcinoma-associated fibroblasts in non-small cell lung cancer. Proceedings of the National Academy of Sciences of the United States of America.

[24] Lili LN, Matyunina LV, Walker LD, Benigno BB, McDonald JF (2013). Molecular profiling predicts the existence of two functionally distinct classes of ovarian cancer stroma. BioMed research international.

[25] Finak G, Bertos N, Pepin F, Sadekova S, Souleimanova M, Zhao H, Chen H, Omeroglu G, Meterissian S, Omeroglu A, Hallett M, Park M (2008). Stromal gene expression predicts clinical outcome in breast cancer. Nature medicine.

[26] Murphy MM, Lawson JA, Mathew SJ, Hutcheson DA, Kardon G (2011). Satellite cells, connective tissue fibroblasts and their interactions are crucial for muscle regeneration. Development (Cambridge, England).

[27] Swierczynski SL, Blackshear PJ (1995). Membrane association of the myristoylated alanine-rich C kinase substrate (MARCKS) protein. Mutational analysis provides evidence for complex interactions. The Journal of biological chemistry.

[28] Chen CH, Thai P, Yoneda K, Adler KB, Yang PC, Wu R (2014). A peptide that inhibits function of Myristoylated Alanine-Rich C Kinase Substrate (MARCKS) reduces lung cancer metastasis. Oncogene.

[29] Chen CH, Cheng CT, Yuan Y, Zhai J, Arif M, Fong LW, Wu R, Ann DK (2015). Elevated MARCKS phosphorylation contributes to unresponsiveness of breast cancer to paclitaxel treatment. Oncotarget.

[30] Ott LE, Sung EJ, Melvin AT, Sheats MK, Haugh JM, Adler KB, Jones SL (2013). Fibroblast Migration Is Regulated by Myristoylated Alanine-Rich C-Kinase Substrate (MARCKS) Protein. PloS one.

[31] Mathew SJ, Hansen JM, Merrell AJ, Murphy MM, Lawson JA, Hutcheson DA, Hansen MS, Angus-Hill M, Kardon G (2011). Connective tissue fibroblasts and Tcf4 regulate myogenesis. Development (Cambridge, England).

[32] Moran-Jones K, Gloss BS, Murali R, Chang DK, Colvin EK, Jones MD, Yuen S, Howell VM, Brown LM, Wong CW, Spong SM, Scarlett CJ, Hacker NF, Ghosh S, Mok SC, Birrer MJ (2015). Connective tissue growth factor as a novel therapeutic target in high grade serous ovarian cancer. Oncotarget.

[33] Cheon DJ, Tong Y, Sim MS, Dering J, Berel D, Cui X, Lester J, Beach JA, Tighiouart M, Walts AE, Karlan BY, Orsulic S (2014). A collagen-remodeling gene signature regulated by TGF-beta signaling is associated with metastasis and poor survival in serous ovarian cancer. Clinical cancer research.

[34] Techasen A, Loilome W, Namwat N, Takahashi E, Sugihara E, Puapairoj A, Miwa M, Saya H, Yongvanit P (2010). Myristoylated alanine-rich C kinase substrate phosphorylation promotes cholangiocarcinoma cell migration and metastasis via the protein kinase C-dependent pathway. Cancer science.

[35] Stensman H, Larsson C (2008). Protein kinase Cepsilon is important for migration of neuroblastoma cells. BMC cancer.

[36] Micallef J, Taccone M, Mukherjee J, Croul S, Busby J, Moran MF, Guha A (2009). Epidermal growth factor receptor variant III-induced glioma invasion is mediated through myristoylated alanine-rich protein kinase C substrate overexpression. Cancer research.

[37] Tan TZ, Yang H, Ye J, Low J, Choolani M, Tan DS, Thiery JP, Huang RY (2015). CSIOVDB: a microarray gene expression database of epithelial ovarian cancer subtype. Oncotarget.

[38] Ganzfried BF, Riester M, Haibe-Kains B, Risch T, Tyekucheva S, Jazic I, Wang XV, Ahmadifar M, Birrer MJ, Parmigiani G, Huttenhower C, Waldron L (2013). curatedOvarianData: clinically annotated data for the ovarian cancer transcriptome. Database.

[39] Tan TZ, Miow QH, Miki Y, Noda T, Mori S, Huang RY, Thiery JP (2014). Epithelial-mesenchymal transition spectrum quantification and its efficacy in deciphering survival and drug responses of cancer patients. EMBO molecular medicine.

[40] Jarboe JS, Anderson JC, Duarte CW, Mehta T, Nowsheen S, Hicks PH, Whitley AC, Rohrbach TD, McCubrey RO, Chiu S, Burleson TM, Bonner JA, Gillespie GY, Yang ES, Willey CD (2012). MARCKS regulates growth and radiation sensitivity and is a novel prognostic factor for glioma. Clinical cancer research.

[41] Neri S, Hashimoto H, Kii H, Watanabe H, Masutomi K, Kuwata T, Date H, Tsuboi M, Goto K, Ochiai A, Ishii G (2016). Cancer cell invasion driven by extracellular matrix remodeling is dependent on the properties of cancer-associated fibroblasts. J Cancer Res Clin Oncol.

[42] Cai J, Tang H, Xu L, Wang X, Yang C, Ruan S, Guo J, Hu S, Wang Z (2012). Fibroblasts in omentum activated by tumor cells promote ovarian cancer growth, adhesion and invasiveness. Carcinogenesis.

[43] Lee KW, Yeo SY, Sung CO, Kim SH (2015). Twist1 is a key regulator of cancer-associated fibroblasts. Cancer research.

[44] Zhang S, Jing Y, Zhang M, Zhang Z, Ma P, Peng H, Shi K, Gao WQ, Zhuang G (2015). Stroma-associated master regulators of molecular subtypes predict patient prognosis in ovarian cancer. Scientific reports.

[45] Winter SF, Lukes L, Walker RC, Welch DR, Hunter KW (2012). Allelic variation and differential expression of the mSIN3A histone deacetylase complex gene Arid4b promote mammary tumor growth and metastasis. PLoS genetics.

[46] Helleman J, Jansen MP, Span PN, van Staveren IL, Massuger LF, Meijer-van Gelder ME, Sweep FC, Ewing PC, van der Burg ME, Stoter G, Nooter K, Berns EM (2006). Molecular profiling of platinum resistant ovarian cancer. International journal of cancer.

[47] Rombouts K, Carloni V, Mello T, Omenetti S, Galastri S, Madiai S, Galli A, Pinzani M (2013). Myristoylated Alanine-Rich protein Kinase C Substrate (MARCKS) expression modulates the metastatic phenotype in human and murine colon carcinoma in vitro and in vivo. Cancer letters.

[48] Yang Y, Chen Y, Saha MN, Chen J, Evans K, Qiu L, Reece D, Chen GA, Chang H (2015). Targeting phospho-MARCKS overcomes drug-resistance and induces antitumor activity in preclinical models of multiple myeloma. Leukemia.

[49] Hartwig JH, Thelen M, Rosen A, Janmey PA, Nairn AC, Aderem A (1992). MARCKS is an actin filament crosslinking protein regulated by protein kinase C and calcium-calmodulin. Nature.

[50] Cao K, Blair CD, Faddah DA, Kieckhaefer JE, Olive M, Erdos MR, Nabel EG, Collins FS (2011). Progerin and telomere dysfunction collaborate to trigger cellular senescence in normal human fibroblasts. The Journal of clinical investigation.

[51] Fruman DA, Rommel C (2014). PI3K and cancer: lessons, challenges and opportunities. Nature reviews Drug discovery.

[52] Polo ML, Riggio M, May M, Rodriguez MJ, Perrone MC, Stallings-Mann M, Kaen D, Frost M, Goetz M, Boughey J, Lanari C, Radisky D, Novaro V (2015). Activation of PI3K/Akt/mTOR signaling in the tumor stroma drives endocrine therapy-dependent breast tumor regression. Oncotarget.

[53] Berdiel-Acer M, Cuadras D, Diaz-Maroto NG, Sanjuan X, Serrano T, Berenguer A, Moreno V, Goncalves-Ribeiro S, Salazar R, Villanueva A, Mollevi DG (2014). A monotonic and prognostic genomic signature from fibroblasts for colorectal cancer initiation, progression, and metastasis. Molecular cancer research.

[54] Huang CR, Lee CT, Chang KY, Chang WC, Liu YW, Lee JC, Chen BK (2015). Down-regulation of ARNT promotes cancer metastasis by activating the fibronectin/integrin beta1/FAK axis. Oncotarget.

[55] Yang Z, Liu Y, Wei X, Zhou X, Gong C, Zhang T, Jin P, Xu S, Ma D, Gao Q (2015). Co-targeting EGFR and Autophagy Impairs Ovarian Cancer Cell Survival during Detachment from the ECM. Current cancer drug targets.

[56] Valencia T, Kim JY, Abu-Baker S, Moscat-Pardos J, Ahn CS, Reina-Campos M, Duran A, Castilla EA, Metallo CM, Diaz-Meco MT, Moscat J (2014). Metabolic reprogramming of stromal fibroblasts through p62-mTORC1 signaling promotes inflammation and tumorigenesis. Cancer cell.

[57] Shen K, Luk S, Hicks DF, Elman JS, Bohr S, Iwamoto Y, Murray R, Pena K, Wang F, Seker E, Weissleder R, Yarmush ML, Toner M, Sgroi D, Parekkadan B (2014). Resolving cancer-stroma interfacial signalling and interventions with micropatterned tumour-stromal assays. Nature communications.

[58] Gyorffy B, Lanczky A, Eklund AC, Denkert C, Budczies J, Li Q, Szallasi Z (2010). An online survival analysis tool to rapidly assess the effect of 22,277 genes on breast cancer prognosis using microarray data of 1,809 patients. Breast cancer research and treatment.

[59] Gyorffy B, Surowiak P, Budczies J, Lanczky A (2013). Online survival analysis software to assess the prognostic value of biomarkers using transcriptomic data in non-small-cell lung cancer. PloS one.

[60] White EA, Kenny HA, Lengyel E (2014). Three-dimensional modeling of ovarian cancer. Advanced drug delivery reviews.

