# Supplementary material for: MARCKS contributes to stromal cancer-associated fibroblast activation and facilitates ovarian cancer metastasis
Source: Oncotarget. 2016 Apr 13;7(25):37649–63. doi: 10.18632/oncotarget.8726 (PMC5122339; doi:10.18632/oncotarget.8726)
Supplement: Supplementary file 1 [file oncotarget-07-37649-s001.pdf]

MARCKS contributes to stromal cancer-associated fibroblast activation and facilitates ovarian cancer metastasis

SUPPLEMENTARY FIGURES AND TABLES

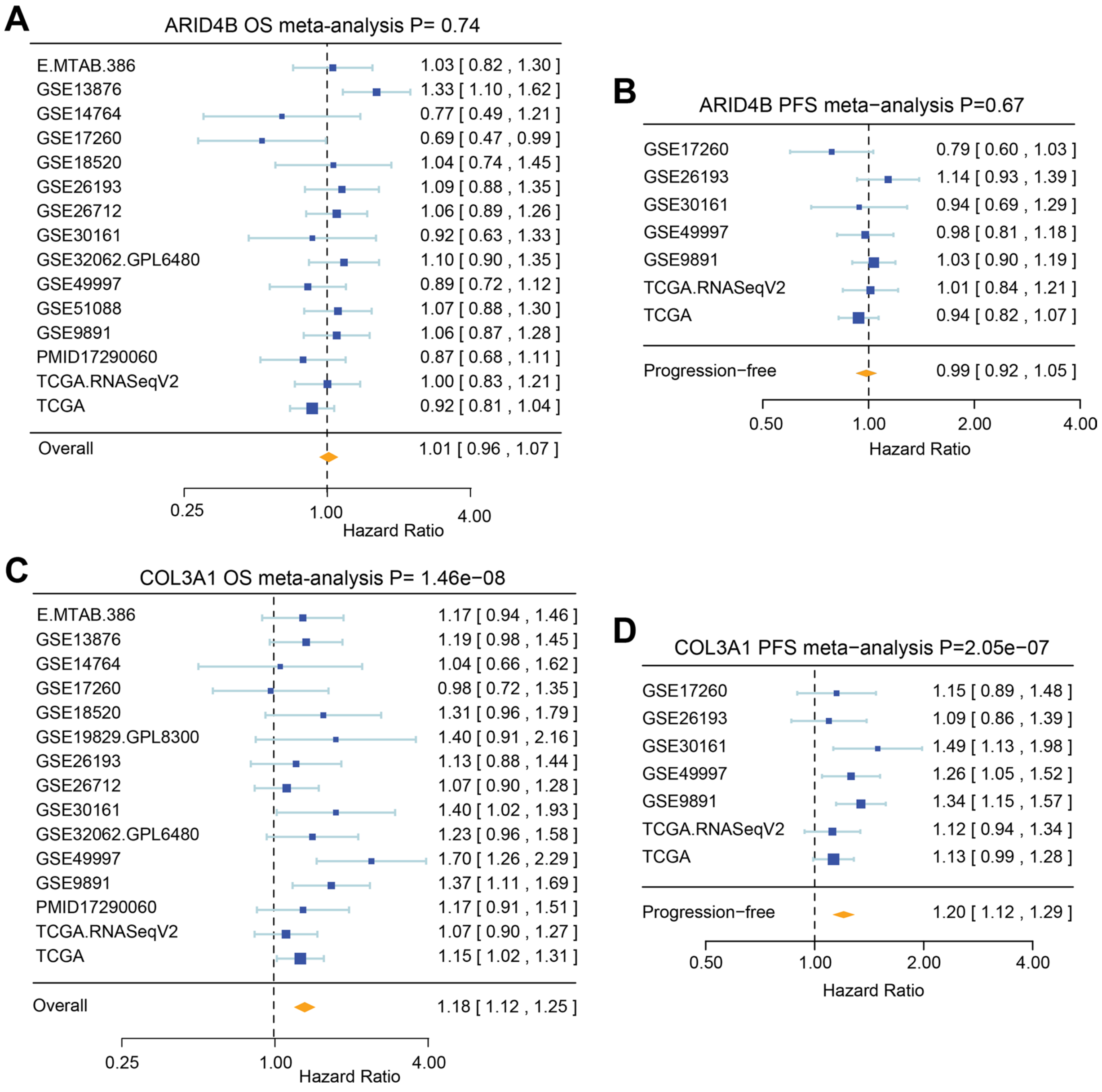

Supplementary Figure S1: The clinical relevance of ARID4B and COL3A1 in epithelial ovarian cancer patients. Meta-analysis depicting the forest plot of ARID4B expression as a univariate predictor of overall survival (OS) **A.** and progression free survival (PFS) **B.** using several datasets with applicable genes expression and survival information of high grade EOC patients. Meta-analysis depicting the forest plot of COL3A1 expression as a univariate predictor of OS **C.** and PFS **D.** using several datasets with applicable genes expression and survival information of high grade EOC patients.

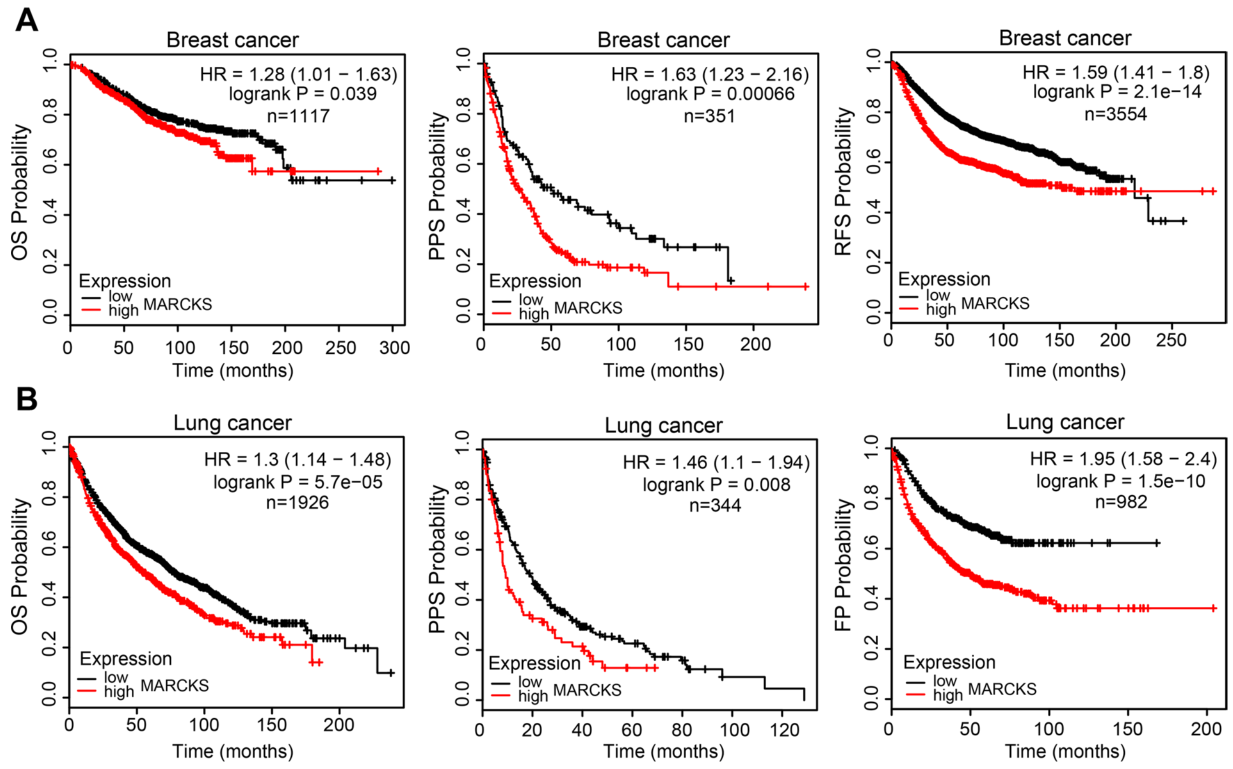

Supplementary Figure S2: Kaplan–Meier analysis for prognostic significance of MARCKS in invasive breast and lung cancer. A. Kaplan–Meier analysis for OS in 1117 cases, post-progression survival (PPS) in 351 cases and relapse-free survival (RFS) in 3554 cases of invasive breast cancer patients according to the mRNA expression of MARCKS over a period of 250 months. **B.** Kaplan–Meier analysis for OS in 1926 cases, PPS in 344 cases and first progression (FP) in 982 cases of lung cancer patients according to the mRNA expression of MARCKS over a period of 250 months.

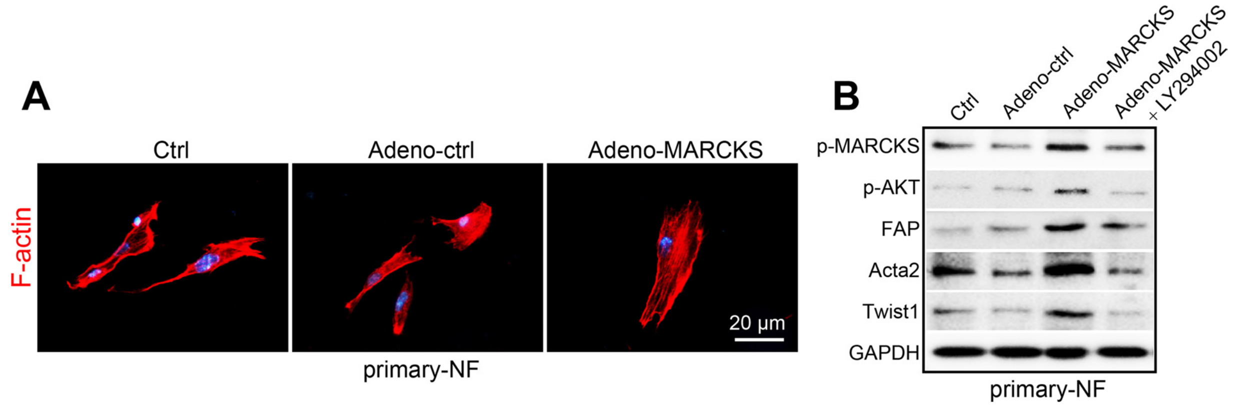

Supplementary Figure S3: Snai2 overexpression induced the CAF phenotype in normal fibroblast involving the activation of the Pi3K/AKT signaling. A. Representative images of F-actin staining in primary normal fibroblast cells after transfection with control adenovirus (Adeno-ctrl) or MARCKS expressing adenovirus (Adeno-MARCKS) for 72 h. **B.** Western blot analysis of p-MARCKS, p-AKT, FAP, Acta2 and Twist1 in primary normal fibroblast cells and primary ovarian CAFs after 72 h treatment with Adeno-ctrl, Adeno-MARCKS in the absence or presence of 2 μM LY294002. GAPDH served as the loading control.

Supplementary Table S1: List of the 503 genes (Score≥0.9) that were calculated strongly positively correlated with ACTA2 expression in dataset GSE40595, which includes profiling data of 39 cases of microdissected normal and ovarian tumor stroma. After selection of the top 25% and the bottom 25% of ovarian stroma samples ranked by ACTA2 expression levels, Gene Set Enrichment Analysis (GSEA) was performed between the two "phenotypes" to determine the genes expression relationship scores regarding to ACTA2 expression and ranked by Pearson's correlation value.

See Supplementary File 1

Supplementary Table S2: List of the 784 probe sets significantly upregulated in ovarian stroma (P<0.05, fold change≥5) compared with corresponding normal ovarian stroma in the GSE40595 dataset, using online analysis tool GCBI website (https://www.gcbi.com.cn) for data normalization and screening of differentially expressed genes based on SAM (Significance Analysis of Microarrays) modified method.

See Supplementary File 1

Supplementary Table S3: List of the 468 probe sets significantly upregulated in invasive breast stroma (P<0.05, fold change≥5) compared with corresponding normal breast stroma in the GSE9014 dataset, which includes profiling data of 59 cases of microdissected normal and breast cancer stroma, using random-variance model (RVM) t test for screening of differentially expressed genes.

See Supplementary File 1
